# Genetic diversity, chemical constituents and anatomical analysis of eight popular Olive (*Olea europaea* L.) cultivars in Al-Jouf region, Saudi Arabia

**DOI:** 10.1038/s41598-024-65542-y

**Published:** 2024-06-26

**Authors:** Haifa A. S. Alhaithloul, Nabil S. Awad, Sameer H. Qari, Rania F. El-Homosy, El-Sayed M. Qaoud, Mesfer M. Alqahtani, Kholoud Z. Ghanem, Abdulrahman Alasmari, Fahad M. Alzuaibr, Hesham S. Ghazzawy, Mohamed A. Abdein

**Affiliations:** 1 Biology Department, College of Science, Jouf University, 2014, Sakaka, Al-Jouf Saudi Arabia; 2 Department of Genetics, Faculty of Agriculture and Natural Resources, Aswan University, Aswân, Egypt; 3 College of Biotechnology, Misr University for Science and Technology, Giza, Egypt; 4 Biology Department, Genetics and Molecular Biology Central Laboratory, Aljumum University College, Umm Al-Qura University, Mecca, Saudi Arabia; 5 Genetics Department, Faculty of Agriculture, Assiut University, Assiut, 71516 Egypt; 6 Horticultural Department, Faculty of Agriculture, Suez Canal University, Ismailia, 41522 Egypt; 7 Department of Biological Sciences, Faculty of Science and Humanities, Shaqra University, P. O. Box 1040, 11911 Ad-Dawadimi, Saudi Arabia; 8 Department of Biological Science, College of Science &Humanities, Shaqra University, 11961 Riyadh, Saudi Arabia; 9 Biology Department, College of Science, University of Tabuk, 47713 Tabuk, Saudi Arabia; 10 Date Palm Research Center of Excellence, King Faisal University, 31982 Al-Ahsa, Saudi Arabia; 11 Central Laboratory for Date Palm Research and Development, Agriculture Research Center, Giza, 12511 Egypt; 12 Seeds Development Department, El-Nada Misr Scientific Research and Development Projects, Turrell, Mansoura, 35511 Egypt

**Keywords:** Olive varieties, ISSR, SCoT, Anatomy, Genetic diversity, Chemical constituents, Biological techniques, Biotechnology, Evolution, Genetics, Molecular biology, Plant sciences, Anatomy, Biomarkers

## Abstract

In light of the multitude of olive trees cultivated and the lack of the genetic diversity of available genotypes to select varieties and lines that are characterized by high diversity and better performance under the corresponding conditions, A comparison analysis of the genotyping and morphological characteristics of eight olive cultivars growing in Saudi Arabia’s Al-Jouf region was conducted and analyzed. Morpho-anatomical and chemical characteristics along with both inter-simple-sequence repeats (ISSRs) and start-codon-targeted (SCoT) markers were used to evaluate the genetic diversity among eight olive varieties in Al-Jouf, Saudi Arabia. Analyses of 27 morphological, chemical, and anatomical characteristics concluded the existence of genetic differences among the studied varieties. Moreover, six ISSR and eight SCoT primer combinations produced a total of 48 loci, of which 18 (10 ISSR and 8 SCoT) were polymorphic. The average polymorphism information content (PIC values of 0.48 and 0.44, respectively) and marker index (MI of 0.79 and 0.48, respectively) detected for ISSR and SCoT markers revealed the prevalence of high genetic diversity among the studied olive varieties. Based on chemical and anatomical characteristics and the selected molecular markers, the eight olive cultivars were grouped into two distinct clusters. Clusters in the adjacent joint dendrogram produced using ISSR, SCoT and combined data were similar, and grouped all individuals into two groups. However, the dendrogram generated on the basis of SCoT separated individuals into subgroups containing at least two varieties. The findings showed that both methods were effective in assessing diversity, and that SCoT markers can be used as a reliable and informative method for assessing genetic diversity and relationships among olive varieties and can serve as a complementary tool to provide a more complete understanding of the genetic diversity available in *Olea europaea* populations in Saudi Arabia.

## Introduction

The Qur’an mentions the olive tree and olive oil seven times^1^; and olives are praised in this text as a precious fruit. The health benefits of the olive tree and olive oil have been described in writings on Al-Tibb Al-Nabawi (prophetic medicine). It was said by Muhammad: “Take olive oil and massage it in, for it is a blessed tree”. Olives are considered a substitute for dates (if dates are not available) during the fasting month of Ramadan, and the leaves of the olive tree are used as incense in some Islamic Mediterranean countries (by burning olive leaves). In fact, archeologists have found significant evidence to indicate that the olive was one of the first trees to be domesticated on the eastern edge of the Mediterranean Basin^2^. Subsequently, olive tree planting spread to the rest of the Mediterranean, thanks to the Phoenician merchants who first brought olive trees to places now synonymous with the production of table olives and olive oil. At present, olives, after the oil palm, are among the most valuable oily fruit trees, covering more than 10 million hectares of land globally^2^. Additionally, olives are grown in many countries and on every continent except Antarctica According to the International Olive Council (IOC), ninety percent of olives are currently designated for oil production, and the other 10% for consumption as table olives^2^. The IOC estimates that about 140 olive varieties grown in 23 diverse countries account for about 85 percent of the world's olive production^3^.

Saudi Arabia depends on the production of olives in the Al-Jouf region of northern Saudi Arabia, near the Jordanian border, where olive trees are widely cultivated. Olive cultivation in the Kingdom of Saudi Arabia first started in Al-Jouf, specifically in the Al-Basitah region, and then widened to the Tabuk region. Al-Jouf began planting olive trees at the onset of 2007^1^. However, 2009 was a turning point in this type of cultivation, when it was expanded to more than 13 million olive trees. There have been significant increases in consumption following changes in modes of food consumption and the great response from consumers, with the annual growth in consumption exceeding that of domestic production at 25 percent, while production does not exceed 10 percent^2^. The Al-Jouf Agriculture Development Company has an area of about 7730 hectares, with 5,000,000 olive trees. Olive leaves are a prolific byproduct of olive trees and can be found in large quantities in the olive oil industry as well as in olive groves^2^. Morph biological characteristics are widely used for descriptive purposes, and are commonly used to differentiate olive genotypes^3^. Agricultural attributes have also facilitated the compilation of different olive cultivars^3^. According to Ref.^3,4^, bio-indexes should be accompanied by a detailed morphological description of the organs (inflorescence, leaves, fruit, and stone) of olive cultivars, following the UPOV method. Several researchers have observed that different varieties are morphologically mutable based on geographic location and different plant growth management practices Ref.^5–7^.

The tasks of a breeding program are to investigate the genetic diversity of available genotypes to select varieties and lines that are characterized by high diversity and better performance under the corresponding conditions^8^. Molecular markers play a major role in estimating diversity and phylogenetic relationships, as they provide notable sources of polymorphism that help breeders to select advantageous traits and thus increase crop yield^9^. Recently, many alternative and effective marker procedures have been created. Start-codon-targeted polymorphism (SCoT) is a novel, simple and reliable genetic marker system. SCoT markers have been used effectively to estimate genetic structure and diversity, and to perform input classification and DNA fingerprinting, in many species^10–21^. This technology incorporates a DNA polymerase chain reaction (PCR)-based DNA marker with several advantages, such as low cost, high polymorphism, and comprehensive genetic information^22^. Additionally, ISSR markers can be efficiently used to assess genetic differences in olive germplasm and genetic similarity and difference between genotypes^23^. The efficiency of ISSR markers is very high, and two primers were sufficient to distinguish some of the varieties examined in a previous study^24^. Moreover, Ref.^25,26^, found ISSR markers to be effective for distinguishing, fingerprinting, and assessing genetic diversity in a collection of genotypes. Polymorphisms of start-codon-targeted (SCoT) markers are reproducible markers based on the short conserved region surrounding the ATG translation start codon in plant genes^27^. Furthermore, Ref ^17,28^. noted that SCoT markers were effective for obtaining new fingerprints for barley and tomato, respectively.

Therefore, the objectives of this study were as follows: (1) to study and compare the polymorphic SCoT and ISSR techniques with high informative values and characterize genetic variation in the selected genotypes that is, to evaluate and analyze the nature and extent of genetic diversity and the genetic relationships among some olive genotypes. (2) To study some of the morphological, biochemical, and anatomical characteristics of olive leaves. (3) To compare and analyze the association of molecular markers with biochemical and anatomical characteristics.

## Materials and methods

### Plant materials

Plant material for eight genotypes Table S. F. (Online Appendix) of mature olive trees planted in Al-Jouf (northern region of Saudi Arabia) were collected by the National Agricultural Development Company (NADEC) and Al-Jouf Agricultural Company during the harvest season of 2021. These cultivars are among those most widely cultivated on a large scale in new orchards in Saudi Arabia, and are highly productive and well adapted to modern olive cultivation techniques. Therefore, they were selected for this study of their molecular diversity.

### Morphometric analysis

Morphometric analysis of the leaves was performed as follows. Length (L), width (W), L/W ratio, and thickness (mm), as well as some micro- and macronutrient contents were measured. Leaf samples were collected from four different trees of each cultivar. The leaves were placed separately in polyethylene bags, placed in an ice box, and transported to the laboratory for refrigeration at − 30 °C and further study. They were cleaned and dried in an electric oven at 70 °C, and ground in a stainless steel mill. The dry powder was digested and analyzed for N, P, K, Na, Mn, and Mg, as described in Ref.^29^.

### Leaf pigment determination

Fresh leaf tissues (0.1 g) were ground in a mill with sand and 70% ethanol solution. The homogenates were then filtered and washed with 70% ethanol (up to 5 ml). After centrifugation for 10 min at 12,100 × *g*, absorbance was read at 646.6 and 663.6 nm for chlorophyll and at 480 nm for carotenoids. The concentrations of chlorophyll a and b (mg/g DW) and total carotenoids (%) were calculated according to Ref.^30^. The total phenol content of the methanolic extract was determined according to Ref.^31^. All measurements were tabulated, and LSD was found among the values of each item corresponding to each trait. Chlorophyll a and b, and total carotenoids and phenols were treated as separate traits, and four traits for eight classes were entered into the system.

### Anatomical studies

To study the anatomical structures of the leaf and stem of the studied cultivars, the leaf or stem samples were fixed in FAA (10 ml formaldehyde: 5 ml acetic acid: 85 ml ethyl alcohol 70%). The selected subjects were washed in 50% ethyl alcohol, dehydrated in ordinary butyl alcohol series, embedded in paraffin wax with a melting point of 56 °C, sectioned to 20 μm thicknesses, double-stained with Safranin and fast green, eluted with xylene, and mounted in Canada Balsam^32–35^. Sections were examined to reveal the histological features of the selected treatments and imaging microscopy was conducted. Clustering was performed on 4 biochemical and 13 anatomical features (6 stem, 7 leaf, separate and combined) using the Euclidean distance matrix and the unweight pair-group method using the arithmetic mean method (UPGMA). The analysis of variance (ANOVA) of the collected data was performed using the SPSS statistical program (SPSS Inc., Chicago, IL, USA) and difference between the means at 5% probability (*p* < 0.05) was considered significant. The statistical differences between treatment means of the data were compared by Least Significant Difference (LSD) test at 5% level of probability (*p* < 0.05). According to Duncan’s test^74^. The software in brackets (link and version number):^75^

https://support.clarivate.com/Endnote/s/article/EndNote-X8-Updates?language=en_US.

### ISSR and SCoT analyses

To achieve the ISSR and SCoT fractions, six ISSR and eight SCoT recommended primers were first selected (Table 1). PCR was carried out in a volume of 25 μl containing master mix beads, 10 μl buffer (10 X), 1 μl primer (100 pmol), 1 μl template DNA (50 ng), and 13 μl sterile HO. Amplification was performed with the cycler programmed as follows: 1 cycle of 94 °C/2 min, 35 cycles (94 °C/2 min, 48 °C/2 min and 72 °C/2 min), 1 cycle of 72 °C/7 min. The degrees of primer annealing varied according to the melting point of each primer. Agarose gel was used to separate the PCR products of the amplified DNA fragments by electrophoresis. An agarose gel was prepared by dissolving 1.2 g agarose in 100 mL buffer including 40 mM Tris acetate and 2 mM NaEDTA.2HO. The gel was stained with ethidium bromide, photographed under UV light, and scanned using a gel documentation system. The data were analyzed using Bio-Rad Model 620 software, USA^25^. Genetic similarity was estimated following^36^. A dendrogram was generated on the basis of similarity matrix data by unweight pair group with arithmetic mean analysis (UPGMA) using the MEGA software 6.0 based on Nei’s genetic distances^37^. In addition, several additional genetic diversity parameters were calculated from the ISSR fingerprinting polymorphism. Informative certainty of primers was analyzed for variation between genotypes by estimating polymorphic information content (PIC) according to Ref.^38^, as PICi = 2fi (1-fi), where PICi is the polymorphic information content of the first i, fi is the frequency of the current bands, and (1-fi) represents the frequency of the absent bands. The highest PIC value for dominant markers is 0.5, according to Ref.^39^. Marker Index (MI) was calculated following^40^, as MI = PIC × number of polymorphic bands.

**Table 1:** Six ISSR and eight SCoT primers used to characterize eight olive cultivars and their corresponding sequences.

No.	ID	Primer sequence (5ʹ → 3ʹ)	ID	Primer sequence (5ʹ → 3ʹ)
	ISSR		SCoT	
1	49A	CAC ACA CAC ACA AG	SCoT 2	CAACAATGGCTACCACCC
2	HB-8	GAG AGA GAG AGA GG	SCoT 3	CAACAATGGCTACCACCG
3	HB-10	GAG AGA GAG AGA CC	SCoT 5	CAA TGG CTA CCA CTA GCG
4	HB-11	GTG TGT GTG TGT TGT CC	SCoT 7	ACA ATG GCT ACC ACT GAC
5	HB-12	CAC CAC CAC GC	SCoT 8	CAACAATGGCTACCACGT
6	HB-13	GAG GAG GAG GC	SCoT 10	ACA ATG GCT ACC ACC AGC
7	----------------------------------		SCoT 12	ACGACATGGCTACCAACG
8	----------------------------------		SCoT 15	CCA TGG CTA CCA CCG GCT

### Plant guideline statement

The plant collection and use was in accordance with all the relevant guidelines.

## Results

**Figure 1:**
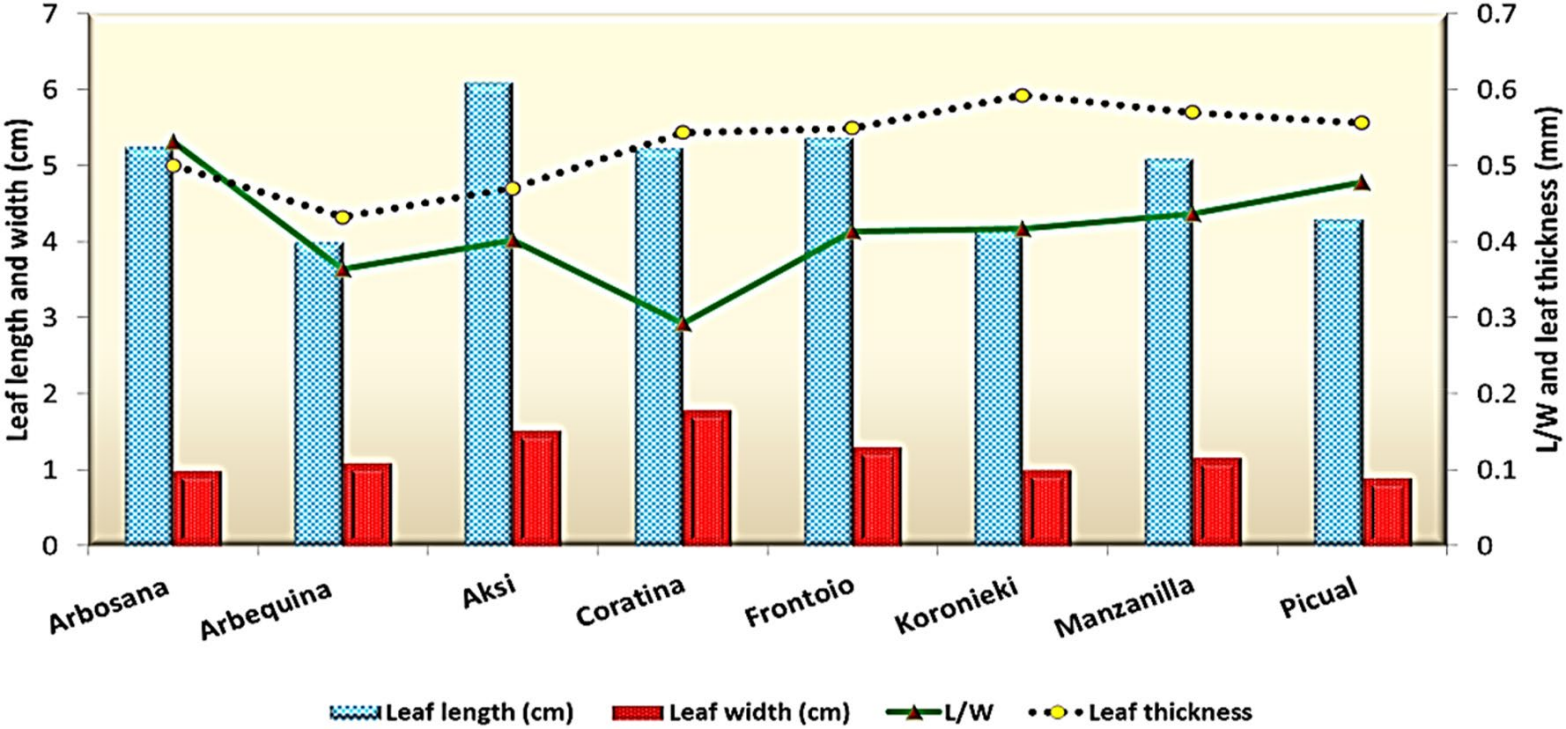
Length, width, length/width ratio, and thickness in leaves of olive cultivars.

**Figure 2:**
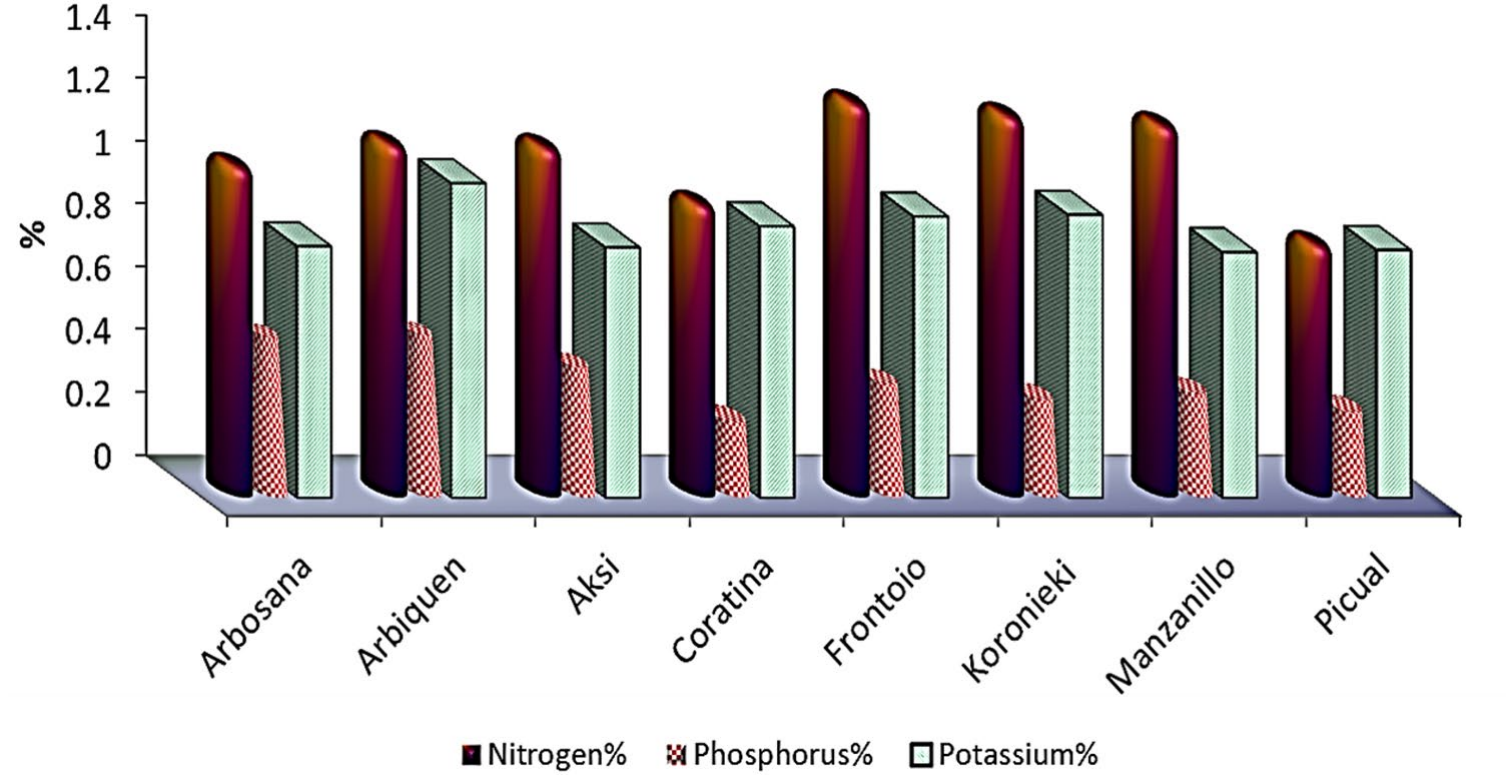
NPK macronutrients in leaves of olive cultivars.

**Figure 3:**
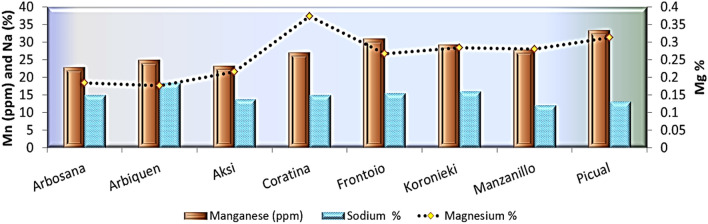
Manganese, magnesium, and sodium nutrients in leaves of olive cultivars.

**Figure 4:**
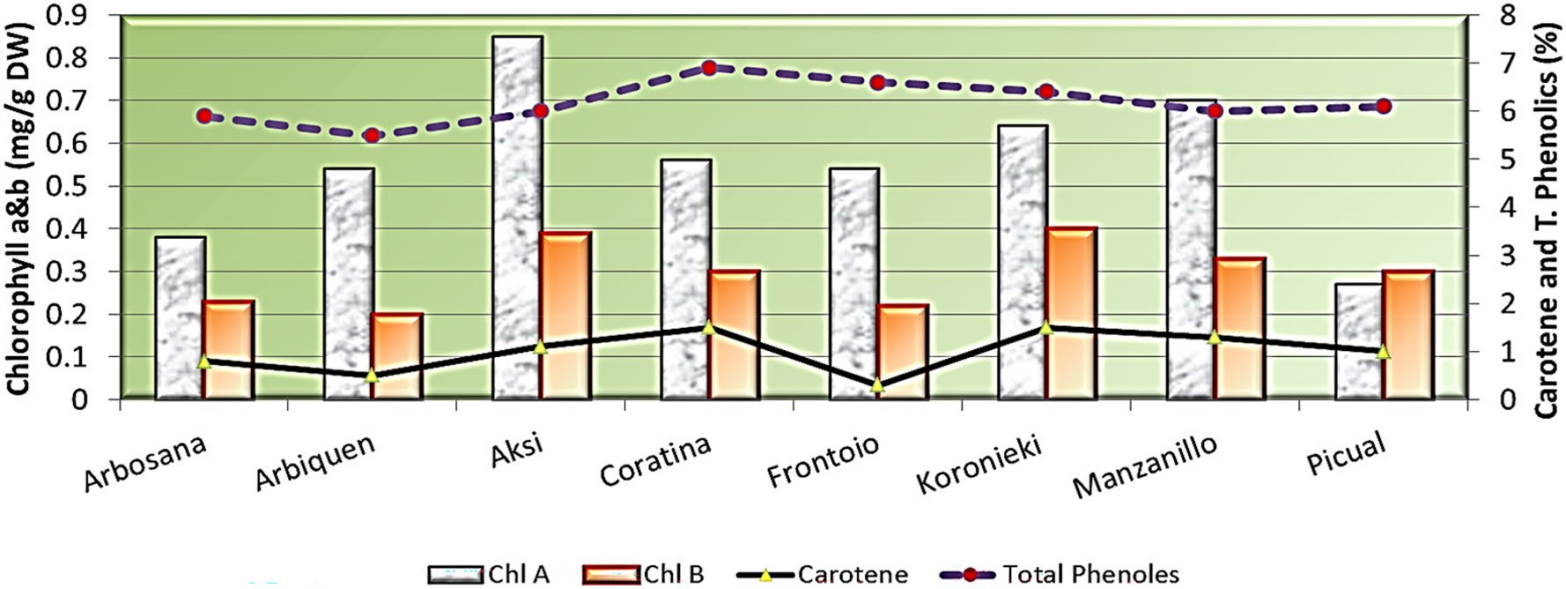
Chlorophyll a and b, carotenoid, and total phenol contents in leaves of olive cultivars.

The analyzed data of 14 horticultural traits of eight olive genotypes are given in Figs. 1, 2, 3, 4 and Table 2. The results showed significant differences between genotypes during the study period for all the studied traits (Table 3). Leaf length values ranged from 4 to 6.1 cm. Among the leaf characteristics, the longest leaf was measured in Aksi, which was on par with Arbosana, Coratina, Frontoio and Manzanillo; however, the shortest leaf was measured in Arbequina. The widest leaf was observed in Coratina, followed by Aksi; the narrowest leaf was in Picual, followed by Arbosana and Koronieki, with no significant differences between them. Leaf shape index was highest in Arbosana (5.31) followed by Picual (4.78), while the lowest was in Coratina (2.93) followed by Arbequina (3.64). The thickest leaf was measured in Koronieki (0.592) followed by Manzanillo and Picual; the thinnest leaf was found in Aksi. Leaf characteristics provide indications of drought tolerance and photosynthesis efficiency.

**Table 2:** Leaf physical characteristics, micro- and macronutrients, pigments, and total phenol concentrations of eight olive cultivars, 2021.

	Arbosana	Arbequina	Aksi	Coratina	Frontoio	Koronieki	Manzanillo	Picual	LSD
Length, width, and shape of leaves									
Leaf length (cm)	5.26	4	6.1	5.24	5.37	4.17	5.1	4.3	1.1
Leaf width (cm)	0.99	1.1	1.52	1.79	1.3	1	1.17	0.9	0.13
L/W	5.3	3.6	4.0	2.9	4.1	4.2	4.4	4.8	0.37
Leaf thickness	0.5	0.432	0.47	0.543	0.549	0.592	0.57	0.556	0.02
N, P, and K concentrations (%)									
Nitrogen%	1.04	1.11	1.1	0.92	1.24	1.2	1.17	0.791	0.03
Phosphorus%	0.493	0.5	0.4	0.23	0.348	0.3	0.32	0.262	0.02
Potassium%	0.8	1	0.796	0.863	0.894	0.9	0.781	0.788	0.04
Mn, Mg, and Na concentrations (%)									
Manganese (ppm)	22.894	25	23.2	27	31	29.314	27.831	33.347	1.16
Magnesium%	0.184	0.176	0.216	0.373	0.267	0.284	0.28	0.313	0.009
Sodium %	14.96	18.41	13.81	14.96	15.54	16.11	12.09	13.24	0.55
Pigments									
Chl A (mg/g DW)	0.38	0.54	0.85	0.56	0.54	0.64	0.7	0.27	0.02
Chl B (mg/g DW)	0.23	0.2	0.39	0.3	0.22	0.4	0.33	0.3	0.05
Carotene (%)	0.8	0.5	1.1	1.5	0.3	1.5	1.3	1	0.18
Total Phenols (%)	5.9	5.49	6	6.9	6.6	6.4	6	6.1	0.1

**Table 3:** Stem and leaf anatomical traits of eight olive cultivars.

Genotypes			G1	G2	G3	G4	G5	G6	G7	G8
STEM	Diameter of	Stem (DS)	3819.9 ± 0.24B	4006.3 ± 0.29A	3769.4 ± 0.29B	4337.9 ± 0.26A	3406 ± 0.25B	4260.5 ± 0.26A	4656.7 ± 0.27A	4402.8 ± 0.26A
		Vascular bundles (DVB)	2515.6 ± 0.12B	2967.9 ± 0.22B	2872.3 ± 0.24B	3010.5 ± 0.25A	2434.5 ± 0.26B	2774.1 ± 0.24B	3486.2 ± 0.26A	2889.8 ± 0.26B
		Pith (DP)	1374.6 ± 0.08	1738.1 ± 0.21	1658.8 ± 0.21	2341.1 ± 0.24	1325.2 ± 0.23	1602.8 ± 0.23	2400.2 ± 0.24	1582.3 ± 0.22
	Thickness of	Cortex (Th.C)	62.7 ± 0.00B	36.8 ± 0.00C	44.6 ± 0.01C	59.2 ± 0.01B	57.1 ± 0.00B	32.9 ± 0.00C	71 ± 0.00A	114.5 ± 0.06A
		Phloem tissue (ThPh)	105.8 ± 0.65B	144.7 ± 0.13B	74.7 ± 0.02C	167.7 ± 0.05A	171.9 ± 0.13A	189.3 ± 0.18A	125.8 ± 0.22B	143.1 ± 0.25B
		Xylem tissue (XT)	173.6 ± 0.34B	263.3 ± 0.22A	219.7 ± 0.12A	253.2 ± 0.07A	215.4 ± 0.23A	246.4 ± 0.16A	147.3 ± 0.23B	161.4 ± 0.16B
LEAF	Thickness of	Lamina (LaT)	363 ± 0.33A	296.9 ± 0.34B	374.9 ± 0.15A	321.7 ± 0.04A	328 ± 0.32A	388.1 ± 0.21A	340.6 ± 0.16A	418.8 ± 0.22A
		Palisade tissue (PT)	115.6 ± 0.21B	104.4 ± 0.03B	131.2 ± 0.04B	97.6 ± 0.01C	124.8 ± 0.22B	126.9 ± 0.12B	125.6 ± 0.10B	177.4 ± 0.11A
		Spongy tissue (ST)	247.5 ± 0.11A	192.4 ± 0.22B	243.7 ± 0.06A	224 ± 0.01B	203.2 ± 0.24B	261.1 ± 0.13A	214.9 ± 0.12A	241.3 ± 0.21A
		Upper epidermis (UET)	12.94 ± 0.24B	16.12 ± 0.11A	18.2 ± 0.01A	17.57 ± 0.00A	23.04 ± 0.00A	16.62 ± 0.00B	9.6 ± 0.00C	14.72 ± 0.00B
		Lower epidermis (LET)	9.99 ± 0.20B	13.19 ± 0.05A	9.6 ± 0.01B	14.31 ± 0.00A	14.94 ± 0.00A	14.51 ± 0.00A	7.68 ± 0.14B	12.43 ± 0.00A
	Mid-vein bundle	Length (L.MVB)	226.3 ± 0.01A	217.7 ± 0.20A	177.2 ± 0.11B	189.5 ± 0.01B	192.15 ± 0.13B	150.2 ± 0.24B	212.2 ± 0.21A	216.9 ± 0.16A
		Width (W.MVB)	298.5 ± 0.3B	331.8 ± 0.2A	312.3 ± 0.13A	375.7 ± 0.02A	288.9 ± 0.27B	281.8 ± 0.24B	369.4 ± 0.22A	352 ± 0.20A

Leaf nitrogen concentration varied significantly between the cultivars. The highest concentration of N in leaves was observed in Frontoio (1.24%), followed by Koronieki (1.2%) and Manzanillo (1.17%), while Picual contained the lowest concentration of N (0.79%) (Table 3). The leaf N concentrations in the cultivars were relatively lower than the critical N level (1.5%). The ability of Arbequina to uptake phosphorous was higher than that of other cultivars (0.5%). The lowest concentration of phosphorous (0.23%) was observed for Coratina. The phosphorous levels of these cultivars were above optimum (0.1%).

The potassium concentrations in the leaves varied significantly (Table 3). The highest K content was observed for Arbequina (1%, 10 g/kg), while Manzanillo and Picual showed the lowest K concentrations (0.78 and 0.79%, respectively).

The cultivars Picual and Frontoio showed the highest concentrations of Mn, with an average of 28.174 mg/kg, while the lowest values were recorded for the Arbosana and Aksi cultivars, with an average of 19.047 mg/kg. However, the concentration of Mn in leaves was above the deficiency level (20 mg/kg) in most cultivars.

As for the chlorophyll content of the leaves, the highest chlorophyll a value was recorded for Aksi, followed by Manzanillo and then Koronieki. The highest chlorophyll b value was also found in Aksi and Manzanillo, with no significant differences between them. Conversely, Picual and Arbequina had the lowest values of chlorophyll a and b, respectively. Coratina and Koronieki cultivars showed the highest contents of carotenoids (1.5%), while the lowest values were recorded for the Frontoio cultivar (0.3%). The Coratina cultivar also showed the highest content of phenols, and Arbequina exhibited the lowest phenol levels.

### Cluster analysis based on phenotypic data

**Figure 5:**
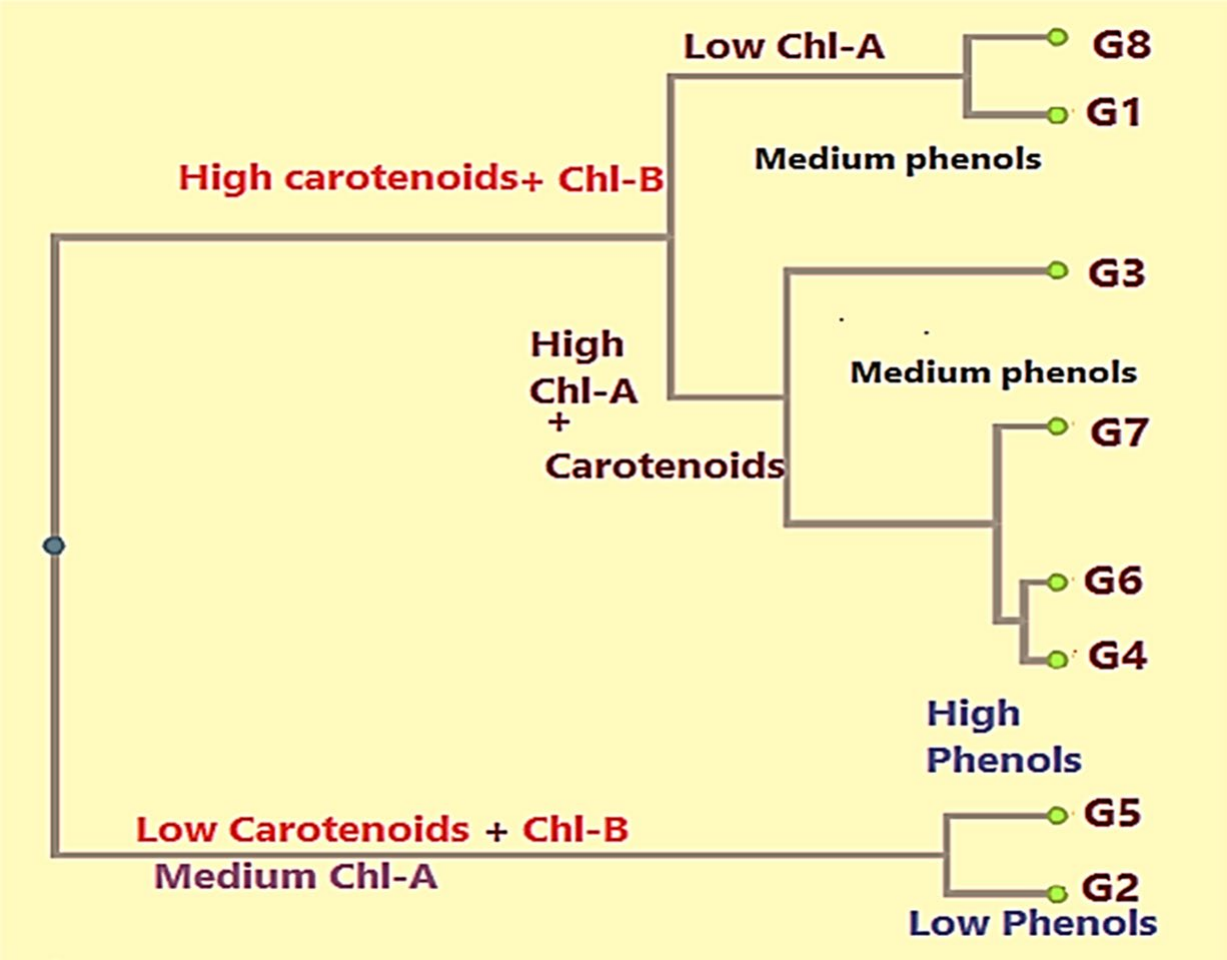
UPGMA clustering dendrogram of olive cultivars based on four biochemical traits.

The clustering pattern of the olive genotypes based on chemical data is depicted in Fig. 5. The analysis assigned the genotypes to two groups. Group 1 included two cultivars characterized by lower levels of carotenoids and chlorophyll b, as well as moderate values of chlorophyll a, and included Frontoio and Arbequina, which had high and low phenol contents, respectively. The second group, which included six genotypes characterized by higher carotenoid and chlorophyll b contents, was separated into two subgroups. Arbosana (G1) and Picual (G8) were grouped together, and had low chlorophyll a and medium phenol contents. The rest of the genotypes (G3, G4, G6, and G7), which had high chlorophyll a and carotenoid contents, were separated into two clusters.

### Anatomical studies

#### Anatomy of the stem

**Figure 6:**
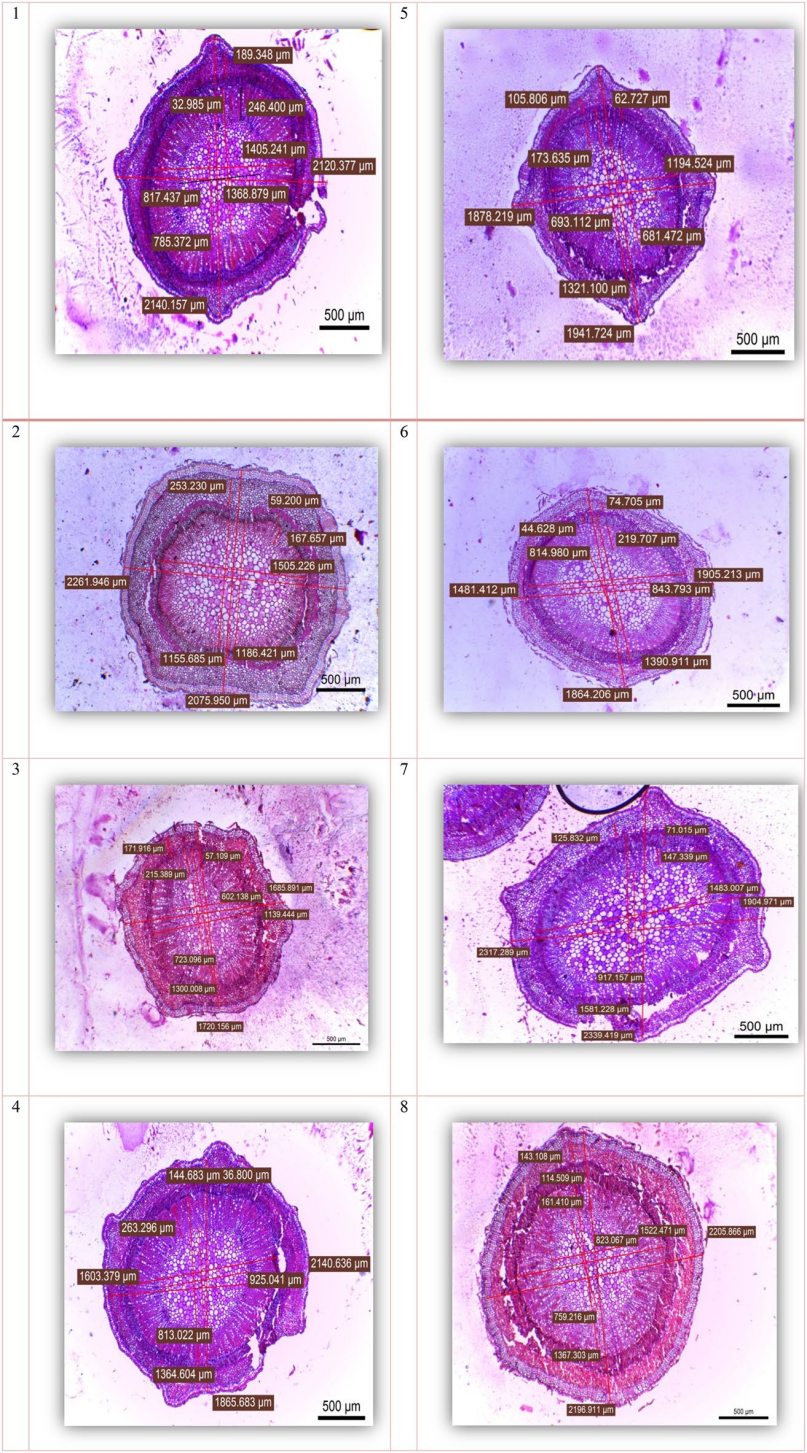
Anatomical structures of the stem of different olive cultivars.

The results presented in Table 3 show that there were significant differences among the cultivars regarding stem tissue parameters, i.e., the diameter of the stem, diameter of the vascular bundles, pith diameter, cortex thickness, phloem tissue thickness, and xylem tissue thickness. The data in Table 4 and the microphotographs shown in Fig. 6 reveal that the Manzanillo cultivar exhibited the highest values for the diameter of the stem, vascular bundles, and pith, followed by the Picual cultivar for stem diameter, which had the highest value for the thickness of the cortex among the different genotypes. Koronieki and Arbequina exhibited the highest thicknesses of phloem and xylem tissue, respectively. Conversely, the Frontio cultivar had the lowest values for the diameter of the stem, vascular bundles, and pith. The Koronieki, Aksi, and Manzanillo cultivars showed the lowest values for cortex thickness, phloem tissue thickness, and xylem tissue thickness, respectively.

**Table 4:** Primer name, total number of bands, number of monomorphic, polymorphic and unique bands, and percentage of polymorphism as revealed by six ISSR and eight SCoT primers for the eight olive cultivars.

Primer code	TAB	Fragment size	MB	PB	UB	P%	PIC	MI
ISSR technique								
49A	3	315:730	2	1	0	33.33	0.47	0.47
HB-8	5	340:765	2	3	2	60.00	0.47	1.41
HB-10	5	245:665	3	2	2	40.00	0.47	0.94
HB-11	4	245:510	3	1	1	25.00	0.50	0.50
HB-12	4	165:470	3	1	1	25.00	0.50	0.50
HB-13	3	370:720	1	2	0	66.67	0.47	0.94
SCoT technique								
SCoT 2	2	230:325	2	0	0	0.00	0.38	0.00
SCoT 3	5	290:815	3	2	0	40.00	0.47	0.94
SCoT 5	4	120:540	2	2	1	50.00	0.50	1.00
SCoT 7	2	500:560	2	0	0	0.00	0.38	0.00
SCoT 8	2	265:330	2	0	0	0.00	0.38	0.00
SCoT 10	3	325:560	2	1	0	33.33	0.47	0.47
SCoT 12	3	285:380	2	1	0	33.33	0.47	0.47
SCoT 15	3	200:615	1	2	1	66.67	0.47	0.94
Total								
ISSR	24		14	10	6	–	2.88	4.75
SCoT	24		16	8	2	–	3.50	3.81
Mean								
ISSR	4		2.33	1.67	1	41.67	0.48	0.79
SCoT	3		2	1	0.25	33.33	0.44	0.48
Max.								
ISSR	5		3	3	2	66.67	0.50	1.41
SCoT	5		2	2	1	66.67	0.50	1.00
Min.								
ISSR	3		1	1	0	25.00	0.47	0.47
SCoT	2		1	0	0	0.00	0.38	0.00

TAB: total amplified bands, MB: monomorphic bands, PB: polymorphic bands. UB: unique bands, PIC: polymorphic information content, MI: marker index; P%: polymorphism (%) for primer.

#### Anatomy of the leaf

**Figure 7:**
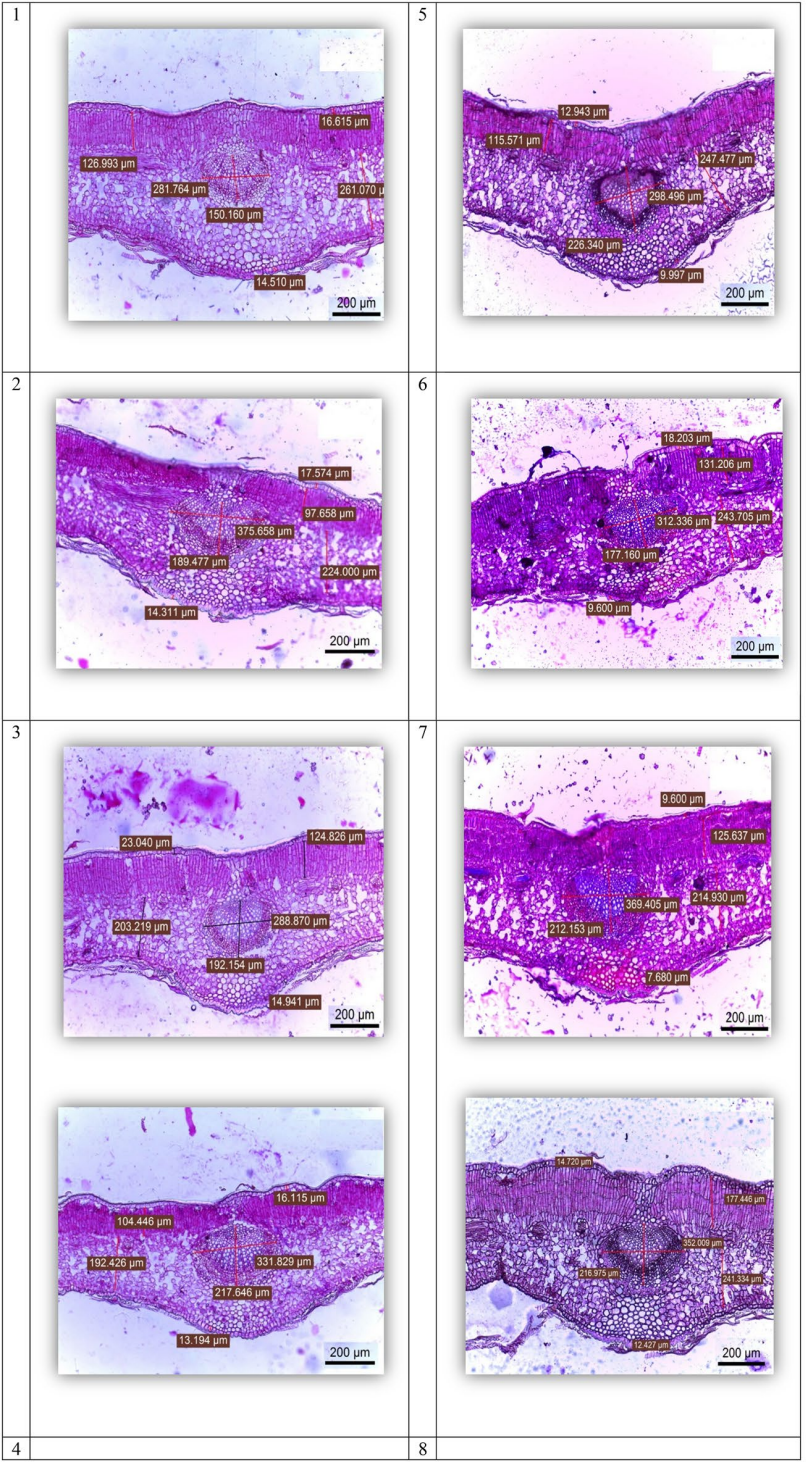
Leaf anatomical structure of different olive cultivars.

The results presented in Table 4 show that there were significant differences between all cultivars with respect to leaf histological characteristics, i.e., lamina thickness, palisade tissue thickness, spongy tissue thickness, mid-vein bundle length, mid-vein bundle width, upper epidermis thickness, and lower epidermis thickness. The data in Table 4 and the microphotographs shown in Fig. 7 reveal that the Picual cultivar exhibited the highest thickness of lamina and palisade tissue, followed by both Koronieki and Aksi cultivars, with the former having the lowest length and width of mid-vein bundle among the genotypes. Arbosana and Coratina exhibited the highest mid-vein bundle length and width, respectively. Conversely, the highest values of both upper and lower epidermis thickness were recorded for the Frontoio cultivar, while Manzanillo exhibited the lowest values for both characteristics.

- According to Duncan’s test. Duncan^74^.

### Cluster analysis based on anatomical data

#### Stem

**Figure 8:**
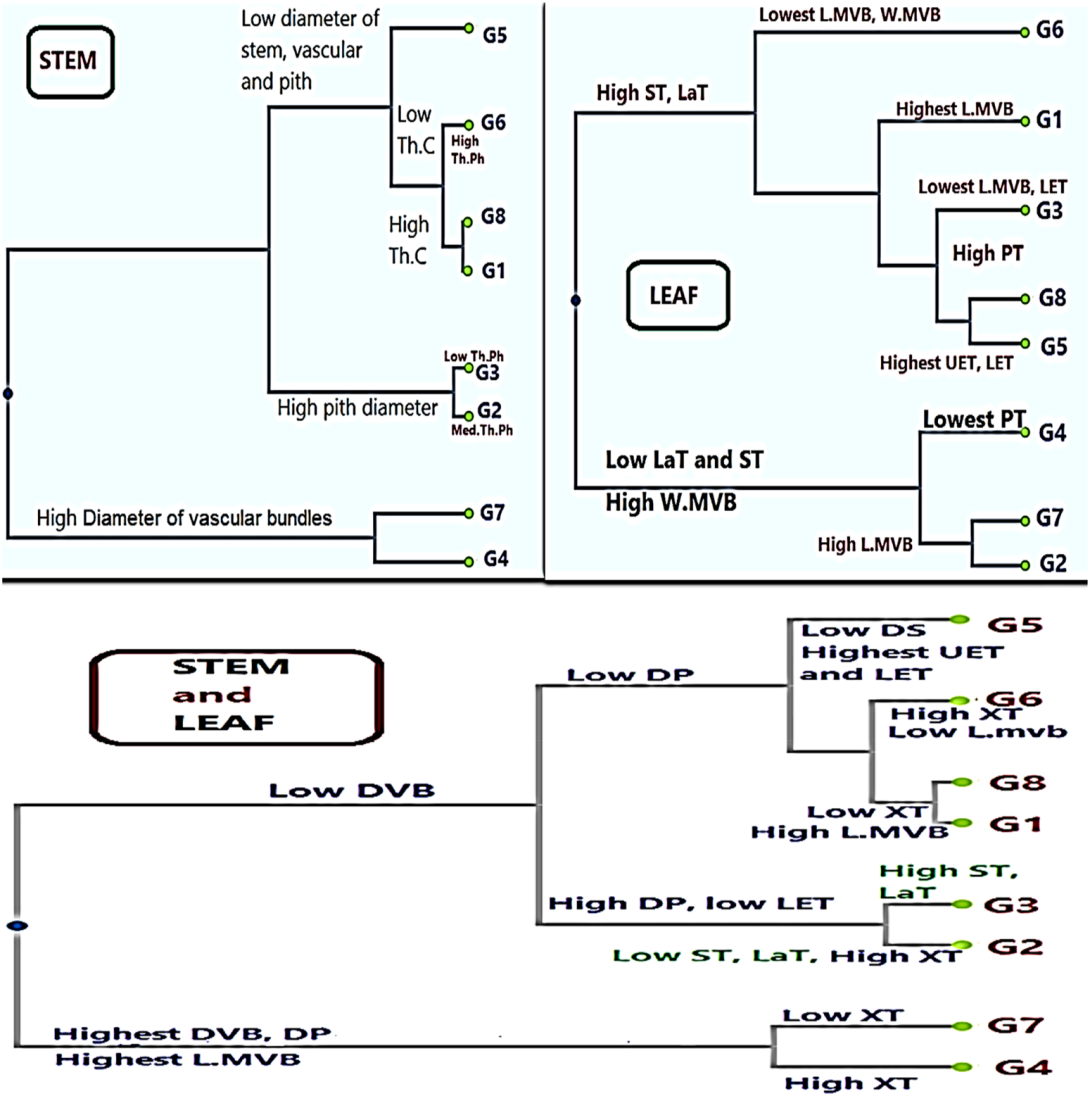
UPGMA clustering dendrogram of olive cultivars based on six stem and seven leaf anatomical traits.

The clustering pattern of the olive genotypes based on stem anatomical data is depicted in Fig. 8 (top left).

This analysis also assigned the genotypes into two groups. Group 1 included two cultivars characterized by higher diameters of both vascular bundles and pith, namely Manzanillo and Coratina. In the second group, in which six genotypes were separated into two subgroups, Aksi (G3) and Arbequina (G2) were grouped together, having higher pith diameters. The other four genotypes were separated into two clusters, where G5, having low diameters of stem, vascular bundles, and pith, was clustered alone. The other three cultivars were grouped together under two sub clusters, with low (G6) and high (G1 and G8) cortex thicknesses.

#### Leaf

The clustering pattern based on leaf anatomical data (Fig. 8, top right) assigned the genotypes into two major groups. Group 1, in which the genotypes were characterized by lower thickness of both lamina and spongy tissue as well as wider mid-vein bundles, was separated into two subgroups, where subgroup I included G4, which had the lowest palisade tissue thickness and mid-vein bundle length values. The other subgroup possessed higher mid-vein bundle length and included both G2 and G7. On the other hand, major group II was characterized by the highest lamina and spongy tissue thickness values and was separated into two clusters, where G6, having the lowest values for both width and length of mid-vein bundles, was clustered alone. The other cluster included two sub clusters, where G1 was clustered alone and the rest of the genotypes were in the other subcluster.

#### Stem and leaf

The clustering pattern of the olive genotypes based on all stem and leaf anatomy characteristics (Fig. 8, bottom) were very close to that based on the stem anatomical traits. The analysis also assigned the genotypes into two groups. Group 1 included two cultivars characterized by higher diameter of both vascular bundles and pith in terms of stem anatomical traits, in addition to having the longest mid-vein bundle in the leaf, namely Manzanillo (thin xylem tissue) and Coratina (thick xylem tissue). The second group contained six genotypes that were separated into two subgroups. Aksi (G3) and Arbequina (G2) were grouped together, possessing the highest pith diameters and having a thin lower epidermis. The other four genotypes, with a lower pith diameter, were separated into two clusters in which G5, with low diameters of stem, vascular bundles, and pith, as well as the highest values of both upper and lower epidermis, was alone in a cluster. The other three cultivars were grouped together under two sub clusters. Low cortex thickness, high xylem thickness, and short length of the leaf mid-vein bundle were found for G6, while the opposite was the case for both G1 and G8, i.e., high cortex thickness, low xylem thickness, and high length of the leaf mid-vein bundle.

## Molecular analysis of markers

### ISSR analysis

**Figure 9:**
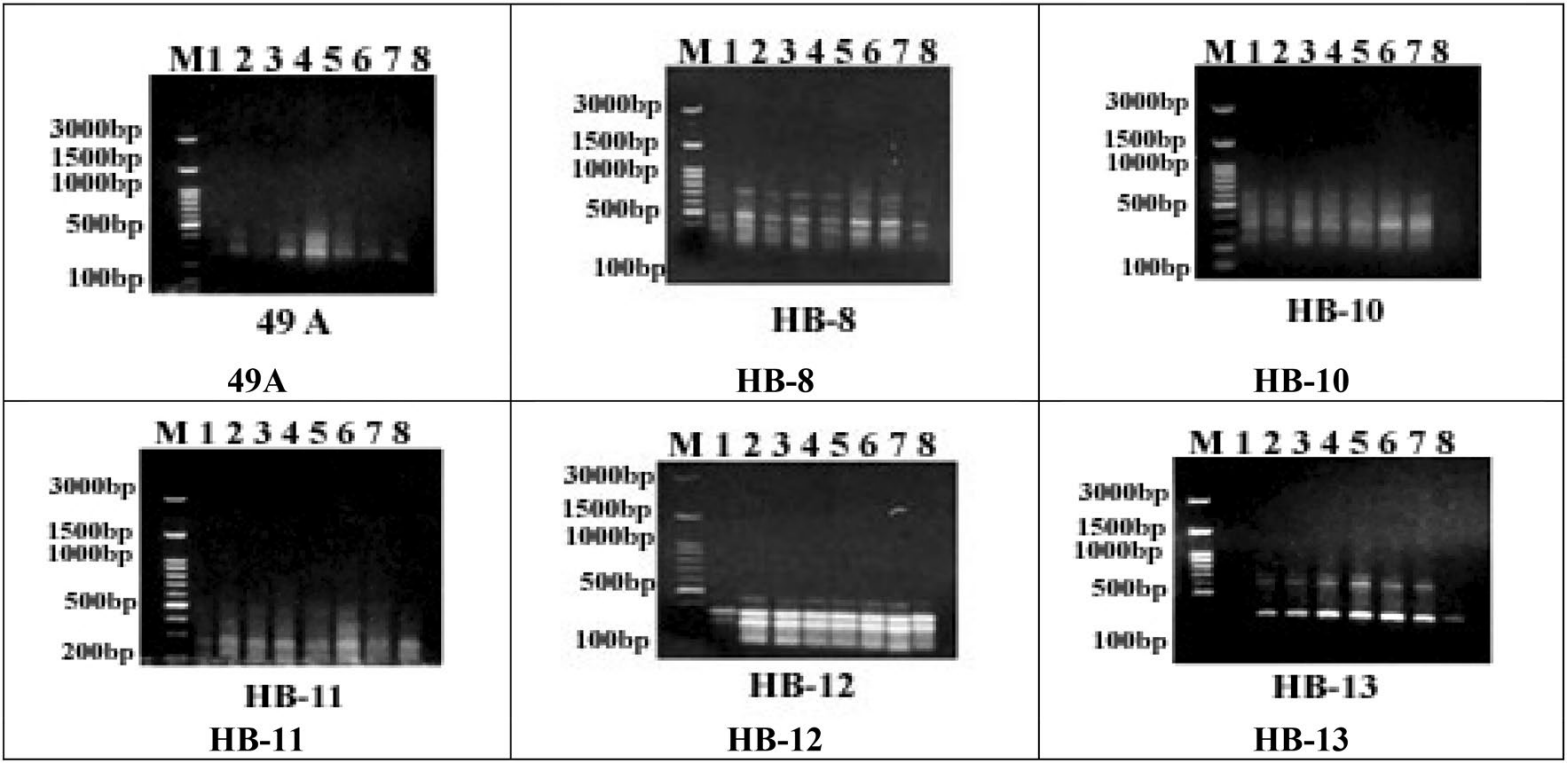
ISSR profile of the eight olive cultivars amplified with six primers.

The generated monomorphic, polymorphic, and unique bands, the percentage and average of polymorphism, as well as the PIC and MI values for the eight cultivars are presented in Table 4 and Fig. 9. In the present study, six selected ISSR primers were used to examine the genetic polymorphism among the eight studied cultivars of *Olea europea*. The obtained data illustrated in Table 4 and Fig. 9 clarify that the size of the amplified bands ranged from about 165 bp (after primer HB-12) to 765 bp (after primer HB-8). The number of bands ranged from five bands (after primers HB-8 and HB-10) to three bands (after primers 49A and HB-13), with an average of four amplicons per primer. All six primers detected polymorphic patterns, revealing a total number of 10 amplicons, with a level of polymorphism of 41.67%.

The highest percentage of polymorphism was 66.67% (after primer HB-13) and the lowest percentage of polymorphism was 25% (after primers HB-11 and HB-12). The number of monomorphic amplicons varied from one (primer HB-13) to three (after primers HB-10, HB-11, and HB-12), with an average of 2.33 monomorphic bands per primer. However, the number of polymorphic bands varied from one (primer 49A, HB-11, and HB-12) to three (primer HB-8), with an average of 1.67 polymorphic amplicons per primer. Moreover, the number of unique fragments varied from zero (49A and HB-13) to two (HB-8 and HB-10), with an average of one unique fragment per primer. The PIC values for the six primers varied from 0.47 to 0.5, with an average of 0.48. Two (HB-11 and HB-12) primers showed 0.5 and the other four showed PIC values of 0.47 (Table 4). Marker index (MI) ranged from 0.47 to 1.41 for the markers 49A and HB-8, respectively.

**Figure 10:**
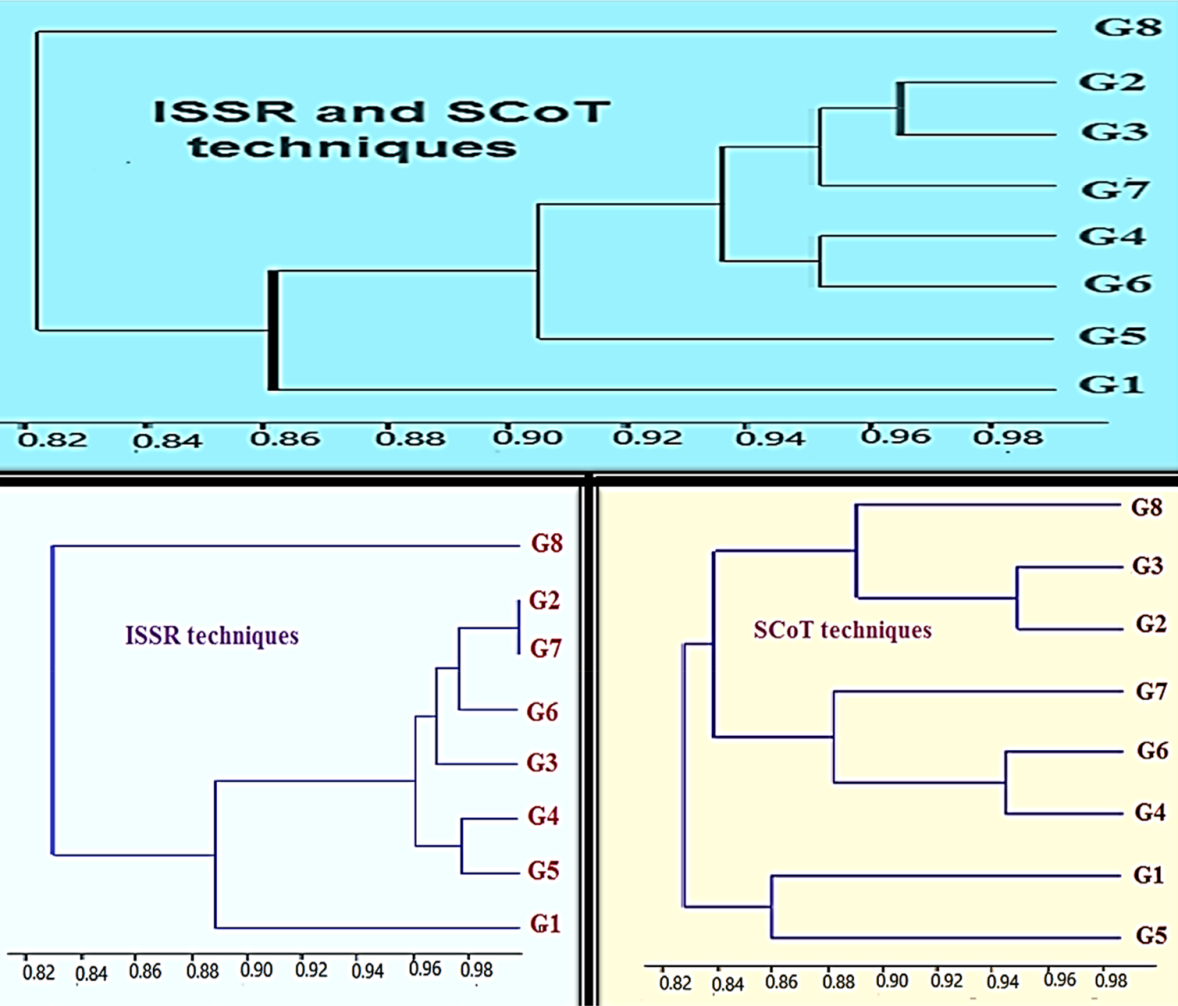
UPGMA dendrogram derived from the genetic similarity within eight *O. europaea* varieties, based on amplification profiles produced by ISSR and SCoT.

Based on the UPGMA clustering algorithm generated from the obtained ISSR data, the eight cultivars were grouped into two clusters (Fig. 10, bottom left), with the Picual cultivar forming a separate cluster. Cluster II was divided into two clades, with the cultivar Arbosana in the first and the second subgroup being divided into two sub clusters. Subcluster I was separated into two clades, with cultivar G4 in the first and G5 in the other. Subcluster II was divided into two clades, with G3 forming a separate clade. The second clade of subcluster II included G6 separately and grouped both G2 and G7 together.

### SCoT analysis

**Figure 11:**
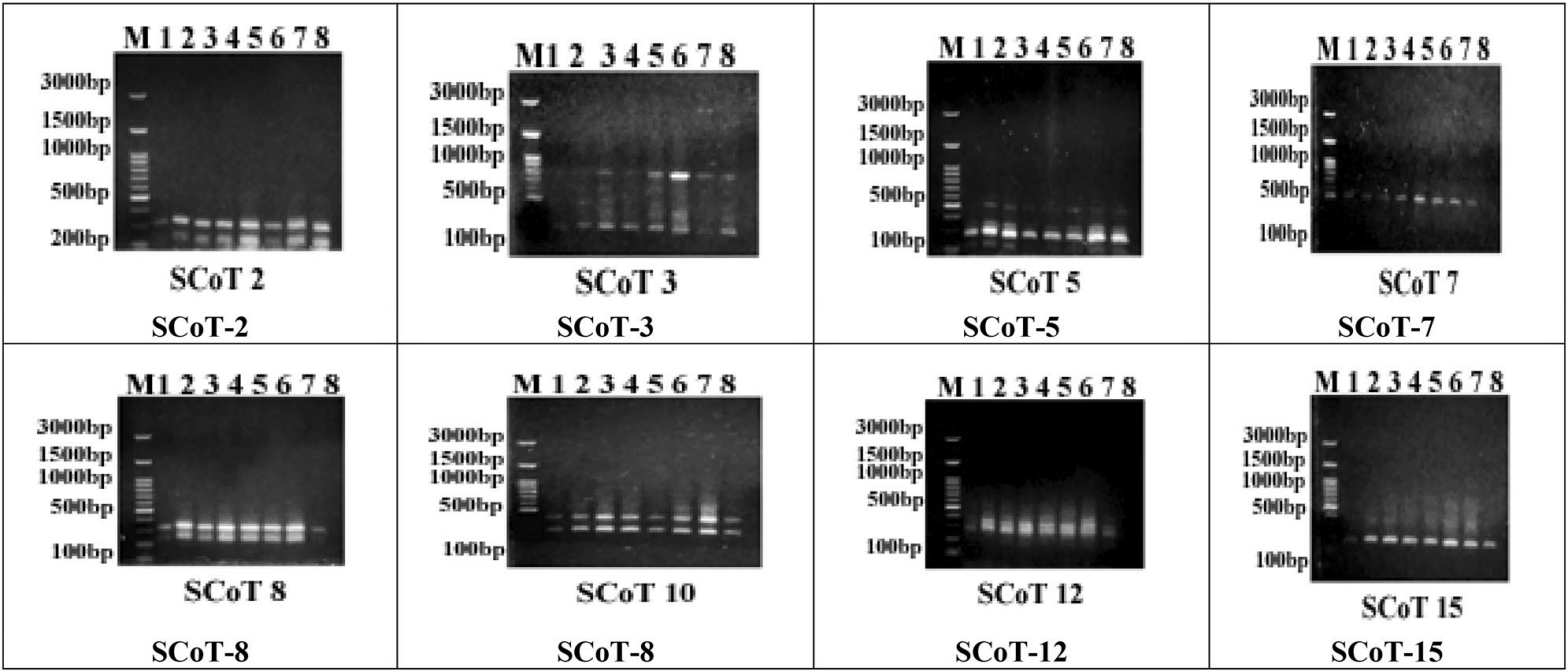
SCoT profiles of the eight olive cultivars amplified with eight primers.

The generated monomorphic bands, polymorphic bands, unique bands, percentage and average of polymorphism in the eight cultivars are presented in Table 4 and Fig. 11. In the present study, eight selected SCoT primers were used to examine the genetic polymorphism among the eight studied cultivars of *Olea europea*. The obtained data illustrated in Table 4 and Fig. 11 clarify that the size of the amplified bands ranged from about 120 bp (after primer SCoT-5) to 815 bp (after primer SCoT-3). The number of bands ranged from two bands (primers SCoT-2, SCoT-7, and SCoT-8) to five bands (after primer SCoT-3) with an average of three amplicons per primer. All eight primers detected polymorphic patterns except SCoT-2, SCoT-7, and SCoT-8, revealing a total number of eight amplicons with a level of polymorphism of 33.33%. The highest percentage of polymorphism was 66.67% (after primer SCoT-15), and the lowest percentage of polymorphism was 0% (after primers SCoT-2, SCoT-7, and SCoT-8). The number of monomorphic amplicons varied from one (after primer SCoT-15) to three (after primer SCoT-3), with an average of two monomorphic bands per primer. However, the number of polymorphic bands varied from zero (primers SCoT-2, SCoT-7, and SCoT-8) to two (primer SCoT-3, SCoT-5, and SCoT-15), with an average of one polymorphic amplicon per primer. Moreover, the number of unique fragments varied from zero (SCoT-2, SCoT-3, SCoT-7, SCoT-8, SCoT-10, and SCoT-12) to one (SCoT-5 and SCoT-15) with an average of 0.25 unique fragments per primer. The PIC values for the eight primers varied from 0.38 to 0.5, with an average of 0.44. The primers SCoT-2, SCoT-7, and SCoT-8 showed the lowest PIC (0.38), and SCoT-5 exhibited the highest one (0.5), as shown in Table 4. Marker index (MI) ranged from 0 to 1 for the markers SCoT-1 and SCoT-5, respectively.

Based on the UPGMA clustering algorithm generated from the obtained SCoT data, the eight cultivars were grouped into three clusters (Fig. 10, bottom right). Cluster I consisted of two clades, with the cultivar Frontoio in the first and Arbosana in the second. The second cluster was divided into two clades, with the cultivar Manzanillo in the first and the second divided into two subclades, with the cultivar Coratina in the first sub-clade and the cultivar Koronieki in the second. Cluster III was divided into two clades, with the cultivar Picual in the first and the second divided into two subclades, with the cultivar Arbequina in the first subclade and the cultivar Aksi in the second.

### ISSR and SCoT combined

On the basis of the UPGMA clustering algorithm generated from the obtained ISSR + SCoT data, the eight cultivars were grouped into two clusters (Fig. 10, top) where the Picual cultivar formed the first cluster. Cluster II was divided into two clades, where the cultivar Arbosana was in the first and the second subgroup was divided into two sub-clusters. Subcluster I contained Frontoio (G5) and subcluster II was divided into two clades, where G4 and G6 formed the first clade. The second clade of subcluster II included G7 separately, and grouped both G2 and G3.

## Discussion

Significant differences were observed in 14 horticultural traits of eight olive genotypes. It is known that leaf characteristics are a sign of high photosynthetic efficiency and drought tolerance, whereby leaf thickness increases when drought occurs, protecting the plant from water loss. A thicker palisade could contain numerous CO_2_-fixation sites, while a thicker spongy parenchyma may result in easier diffusion of CO_2_ to these sites^41^. Genetic differences in leaf morphology and anatomy form a basis for comparison, particularly with respect to differences in drought resistance, between any two cultivars. The structural features of leaves can be considered to govern the ability of a tree to resist water stress. Therefore, they can be used as criteria for selecting olive cultivars for resistance to drought and high photosynthetic efficiency^41,42^. As for NPK macro nutrients, leaf N concentration was significantly affected by cultivar. The Frontoio cultivar exhibited the highest N concentration (1.24%) in the leaves, whereas Picual contained the lowest N (0.79%). Many researchers have demonstrated that N is transported to fruits, and that leaf N content is decreased in “on” years^34,43–49^. The results revealed that the leaf N concentration of the cultivars was relatively lower than the critical N level (1.5%)^50,51^. On the basis of experiments conducted in Greece and Portugal, reported that leaf N concentrations of different olive cultivars were above the critical level. Conversely, Ref.^52^ found comparatively higher values for leaf N concentration within the range of 16.8–28.9 g/kg. On the other hand, although the cultivars that accumulate greater amounts of nitrogen are usually those with high uptake efficiency, the relationship between nitrogen uptake efficiency and plant nitrogen content is not clear, and these parameters are not related to plant strength, indicating that those plants that grow more are not necessarily those that are most efficient at accumulating or absorbing nitrogen^53^.

The ability of Arbequina to uptake phosphorus was higher than that of other cultivars (0.5%). The lowest P concentrations (0.23%) were observed for Coratina. Ref.^43,49^ concluded that the P content of leaves was comparatively reduced in “on” years, due to its transport to the fruits. The phosphorus levels in the leaves of cultivars were above the optimum level (0.1%). A much higher range of P concentrations (0.17–0.27%) was reported previously for mature leaves^52^. In contrast, leaf P concentrations in Greek and Portugese olives were in the ranges of 0.13–0.16% and 0.12–0.19%^48,51^, respectively.

The K concentration of the leaves varied significantly. The highest K content was observed in Arbequina (1%, 10 g/kg) whereas Manzanillo and Picual showed the lowest K concentrations (0.78 and 0.79%, respectively). In the studies presented in Ref.^43,48,54^, it was found that K was negatively correlated with yield, and increased yield and increased K transfer to fruit led to a decrease in leaf K content. Ref.^55^ showed that potassium concentration ranged between 0.72 and 1.0%, and lower potassium concentrations were observed in cultivars with higher oil content. K deficiency is a common problem in no irrigated olive orchards^56^, due to the inhibition of K uptake; the availability of K in the soil decreases with lack of irrigation. Additionally, more than 50% of Turkish olives in “on” years have been reported to suffer from K deficiency problems in many regions^55–57^ in which rain-fed farming is practiced, despite the soils having a very high content of exchangeable potassium^58^ Leaf K concentration has been reported to be 5.0–9.0 g/kg in Ref.^59^ and 5.4–8.3 g/kg in Portugal^40^.

In the study presented in Ref.^55^, the measured K concentrations were relatively higher due to the higher K exchange content in the experimental soil (395–500 mg/kg) and the potassium-uptake-inducing irrigation. As for micronutrients, the Picual and Frontoio cultivars showed the highest concentrations of manganese, with an average of 28.174 mg/kg, while the lowest values were observed for the Arbosana and Aksi cultivars, which showed an average of 19.047 mg/kg. These results are consistent with Ref.^55^, where it was found that Mn concentration was inversely related to yield, regardless of cultivation. However, the Mn concentration in the leaves was much higher than the decline rate (20 mg/kg) in cultivation. In agreement with what was found in this study^48^ reported low concentrations of manganese in “current” years. The varieties used in this study contained less manganese than the Greek olives (33–72 mg/kg), but were similar to the Portuguese olives (14.9–33.7 mg/kg). The concentrations of manganese in leaves range from 14.0 to 52.8 mg/kg^42^ with seasonal variation.

The UPGMA dendrogram, based on the similarity matrix for ISSR and SCoT and created using composite data, shows the similarities between the eight types, and is composed of two of the three main groups. Moreover, the dendrogram based on the data for the chemical traits (chlorophyll, carotenoids, and phenols) clearly separates Arbequina and Frontoio into a separate cluster, leaving the six remaining cultivars to form two groups in the second cluster. The first group was composed of the cultivars Picual, Arbosana, and Aksi. Interestingly, the cultivars Arbosana and Picual were the closest. Moreover, the last group contained the cultivars Coratina, Koronieki, and Manzanillo. These findings are congruent with the results presented in Ref.^60,61^, where it was found that Aksi and Picual were clustered in one independent group and Koronieki was in another sub-group in an analysis of 32 *O. europaea* varieties using SSR and ISSR markers. On the other hand, the ISSR markers and the combined data divided Picual into a cluster on its own on the dendrogram. Although Arbequina and Oxy were included in Cluster III by SCoT, Frontoio and Coratina were successfully clustered in the dendrogram of the ISSR data. The same trend was reported by Ref.^62^ for the groups between the Italian Frontoio and Coratina varieties, using a UPGMA dendrogram on the basis of the data for AFLP and SSR markers, as well as^17^ by using a UPGMA dendrogram on the basis of data for SCoT and ISSR markers.

Remarkably, the Coratina and Koronieki cultivars and the Arbequina and Aksi cultivars had the tendency to form separate corresponding groups when using SCoT markers for all anatomical stem + leaf traits for Coratina and Koronieki and chemical markers for Arbequina and Aksi; these groups were further confirmed by the ISSR + SCoT combined dendrogram. Additionally, all markers successfully isolated Manzanillo from Koronieki into separate clades or clusters. These results, with respect to the genetic similarity revealed by different marker types, are attributed to the different mechanisms that identify the polymorphism and genetic coverage associated with each marker. Therefore, the genetic similarity of the US data may be more representative of the genetic relationship. Similar investigations have been reported in the past^61,63,64^.

The use of ISSR and SCoT enabled a better comparison of the effect of each gene marker in classifying olive varieties. These markers successfully applied a unique fingerprint to each olive variety using six ISSR and eight SCoT primer combinations, resulting in a total of 48 reproducible bands (24 each), with percentages of polymorphism of 41.67% and 33.33%, respectively. The polymorphism % produced by these markers revealed that the olive tree is a highly polymorphic species, reflecting the agronomic diversity within olive cultivars. Polymorphism rates have previously been recorded in Ref.^65^ (61%), Ref ^66^. (36.14%) and Ref ^67^. (38.22%). These variations can be attributed to differences in genotypes. These results are in accordance with the findings obtained in Ref.^61,63,64,68,69^. The specific power and usage of each marker was estimated by comparing its PIC and MI values. The ISSR markers demonstrated a higher value of polymorphism compared to SCoT, with a PIC value of 0.48 and MI of 0.79, indicating that these loci were the most informative for the olive cultivars studied^70^. The primers were identified. Those with a PIC value of 0.5 > PIC > 0.25 were considered to be informative markers. Of the two molecular marker types used, the ISSR markers were the most discriminating, providing more informative data than the SCoT markers. ISSR markers can therefore be used as an effective integral method compared to SCoT for determining the molecular properties and genetic relationships of olives. This finding coincides with Ref.^71^ where it was reported that the average percentage of polymorphism using SCoT markers was more informative and effective for fingerprinting than other markers in terms of PIC. Consequently, these results are consistent with previous studies^22,72,73^ in which PIC, EMR, and MI were considered to be the most important parameters for the selection of informative markers. The results indicated that SCoT markers could be used as an effective complement to ISSR, because SCoT are genetically targeted markers and can effectively generate trait-associated markers and be used to study genetic relationships. Based on the results, a molecular database can be created for olive identification quality indices and evaluation of olive oil^76^. A molecular catalog can be used to identify molecular specimens of cultivars and make a reference group to exclude unnecessary genetic factors.

## Conclusion

Genetic diversity assessment using molecular markers is still one of the most important study types and most efficient ways to investigate the variation and diversity between some economically significant *O. europaea* varieties. ISSR and SCoT markers are powerful tools for *O. europaea* varietal identification as well as for accurately establishing traits of the eight varieties examined. Thus, the present results could be used to construct a molecular marker database for *O. europaea* identification and accurate molecular maps for *O. europaea* varieties. Moreover, they offer a better understanding of the variability available in the *O. europaea* varieties grown in Saudi Arabia. The results of this study can offer information for reliable *O. europaea* breeding programs and Vision 2030 in Saudi Arabia.

### Supplementary Information

Supplementary Information.

## Data Availability

The datasets used and/or analyzed during the current study are available from the corresponding author on reasonable request.
