# Supplementary material for: Genetic diversity, chemical constituents and anatomical analysis of eight popular Olive (Olea europaea L.) cultivars in Al-Jouf region, Saudi Arabia
Source: Sci Rep. 2024 Jun 26;14:14688. doi: 10.1038/s41598-024-65542-y (PMC11199486; doi:10.1038/s41598-024-65542-y)
Supplement: Supplementary file 1 — Supplementary Information. [file 41598_2024_65542_MOESM1_ESM.docx]

Appendix

Table S.F: Description of the eight studied olive cultivars.

| Description of the eight studied olive cultivars |
| --- |
| Arbequina: The second most used olive oil after Picual. Small brown olives, native to Spain, good for eating and oil. The highly aromatic fruit is at first green and matures into brown or completely brown. Expect heavy yields and high yields of oil. A very compact tree, good for intensive planting and small spaces, the fruits are round, weighing 1-2 grams. The core is smooth and loose from the pulp, making up 16% of the weight of the fruit. Oil content of 17-20%. Arbequina olives are easily harvested and are frequently selected for automated harvesting processes. Its high oil content and adaptability contribute to its popularity among producers. |
| Manzanillo: A hardy plant with good growth. The most important Spanish variety. It is appreciated in international markets for its distinctive fruit shape, ease of pit removal, and excellent pulp quality. The leaves are medium–small, oval in shape, and bright green in color. Manzanillo or Manzanilla; "Manzanillas" means "little apple" in Spanish. A large, round, oval-shaped fruit, famous for its rich taste and thick pulp. It is a prolific bearer, and it grows all over the world. Its skin is a glossy purple, turning dark blue upon maturity. Resists bruising and matures early. The pulp portions easily with its bitterness and is extremely rich when pickled. Excellent for oil and pickles. A spreading and powerful tree. |
| Frantoio: Constantly produces high-yield fruit oil. The tree grows moderately and has a well-ventilated canopy. They tend to be very productive in the right conditions and tend to grow more like a tree than a shrub, in contrast to most olive trees. The average oil yield is 23-28% from the fruit. A self-compatible tree, but would greatly benefit from other pollinators. |
| Koroneki: Originated in the southern Peloponnese, around Kalamata and Mani, in Greece. Although this young olive is difficult to grow, it has a high yield of olive oil of exceptional quality. Additionally known as Koroni, Kritikia, Ladolia, Lianolia, Psilolia, and Vaciki (Olea Database: Koroneiki). The oil has a very light and harmonious aroma, often with a slight lemon scent. Together with the Arbequina and Picual varieties, Koroneiki are well suited for intensive (mechanical high-density) harvesting. Koroneki olives are grown in 19 different countries around the world. In general, Koroniki olives are high in polyphenols and oleocanthal, which gives the oil a strong, bitter flavor. |
| Picual: The most important variety in Spain, distinguished by excellent characteristics of olive oil production (high-performance olives, high yield capacity, and ease of separation). About a third of the world's olive oil production comes from Picual olives. With supportive watering and proper pruning for training, a Picual olive tree takes about 5-6 years to produce good crops. It represents about 50% of olive production in Spain and about 20% of olive production globally. It has a strong but sweet flavor, and is widely used as a table olive. |
| Arbosana: Matures very early (it begins producing harvests after only two years) and is very productive. With proper management, it is possible to reach average productions greater than 2000 kg of olive oil per hectare. It has good characteristics for producing olive oil in terms of quantity and quality, and is a medium-ripening olive  (harvested three weeks after Arbequina). |
| Coratina: Trees produce large, round olives with an oil yield of up to 25 percent. The oil is generally strong and bitter, and is also touted for its high level of polyphenols and other antioxidants. It is one of the most important Italian cultivars and is also available in Argentina, Australia, and northern California. These olives contain a high level of polyphenols naturally, resulting in a strong-tasting olive oil. |
| Aksi (Agizi-Aksi): Egyptian local variety is spread in Fayoum, Egypt, mixed with Al-Ajizi Al-Shami and it is very similar except that the fruit is smaller, weighing 6-8 grams, with a wide base and a pointed head with a slight bend. The fruits are used in green pickles and ripen from October to December. |

*The software in brackets (link and version number):

https://support.clarivate.com/Endnote/s/article/EndNote-X8-Updates?language=en_US

**Steam Olive**

| 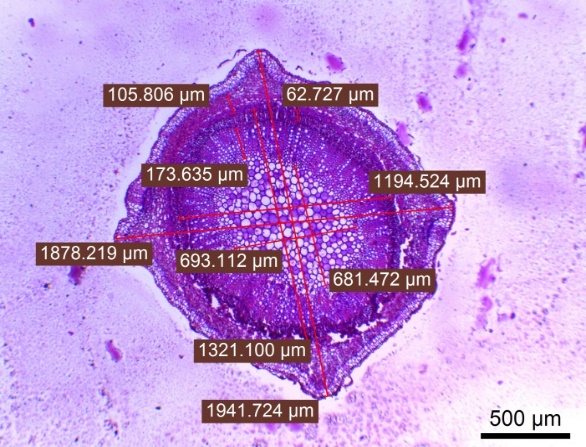 | 5 | 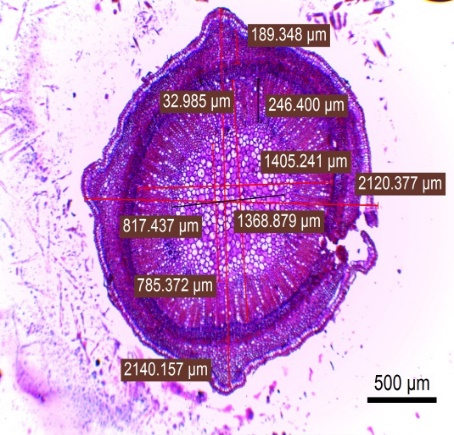 | 1 |
| --- | --- | --- | --- |
| 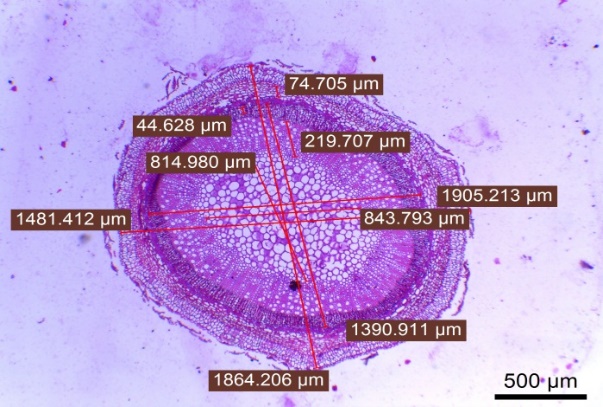 |  | 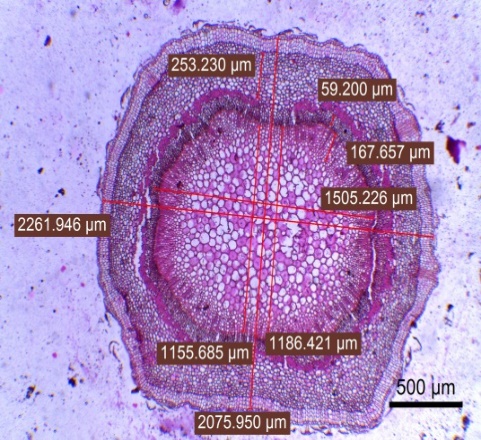 | 2 |
| 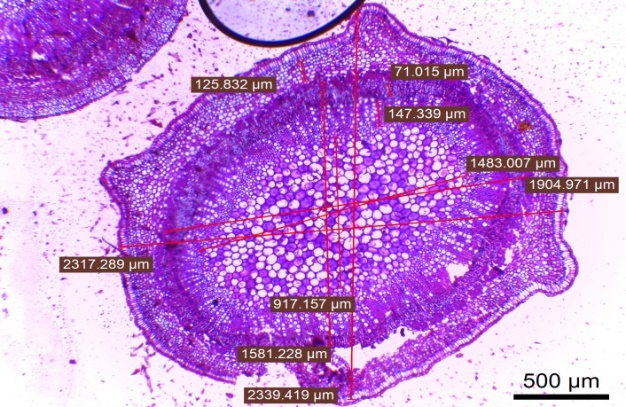 | 7 | 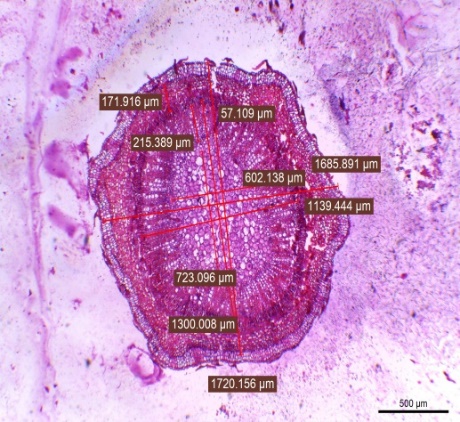 | 3 |
| 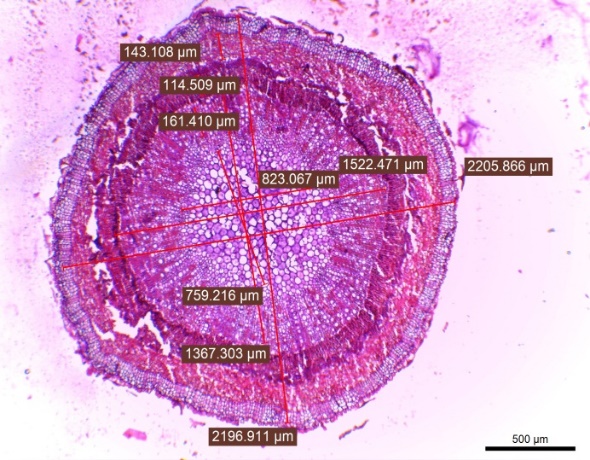 | 8 | 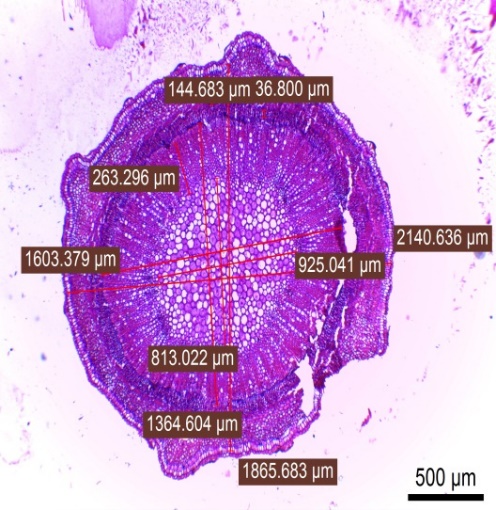 | 4 |

Figure 6. Anatomical structures of the stem of different olive cultivars.

**Leave Olive**

| 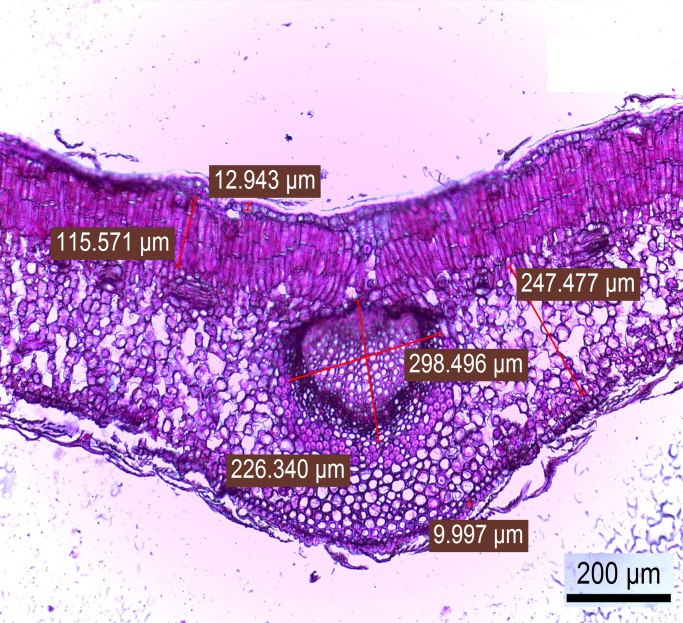 | 5 | 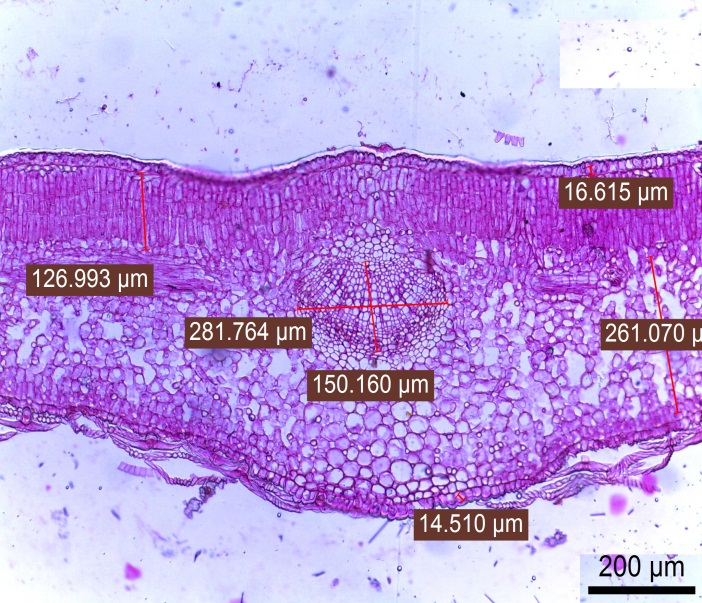 | 1 |
| --- | --- | --- | --- |
| 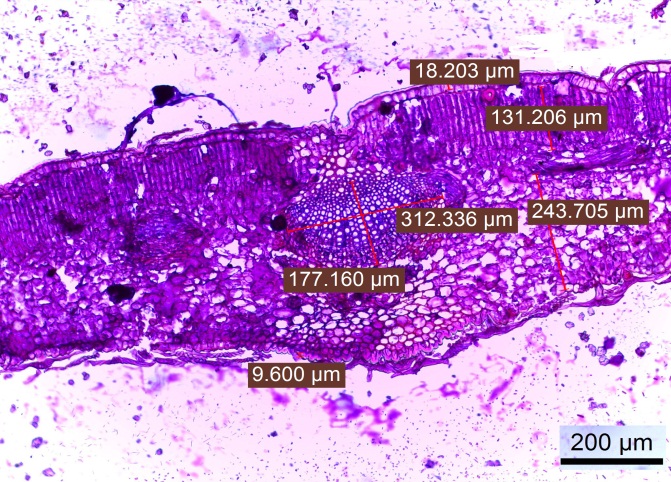 | 6 | 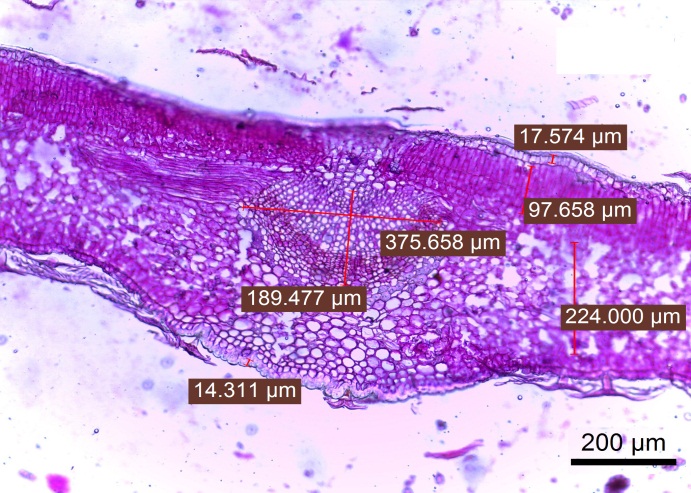 | 2 |
| 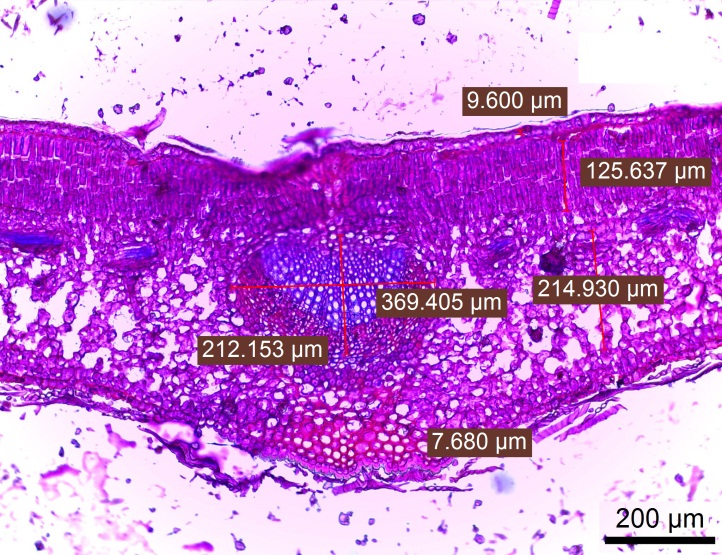 | 7 | 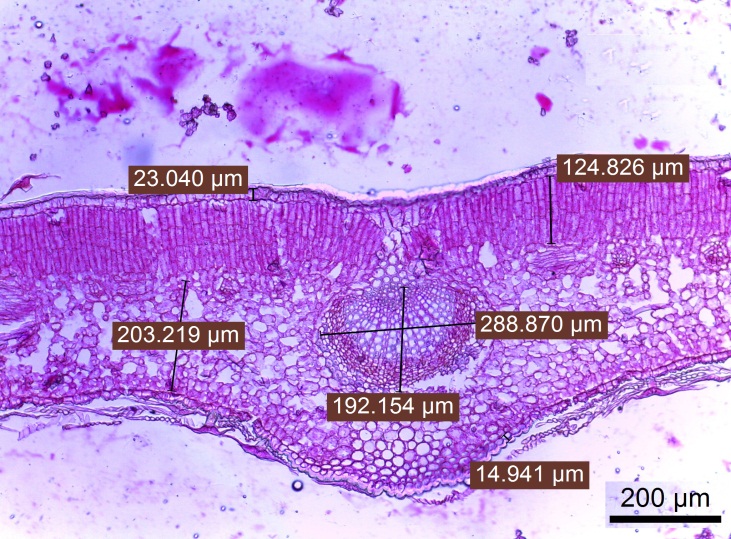 | 3 |
| 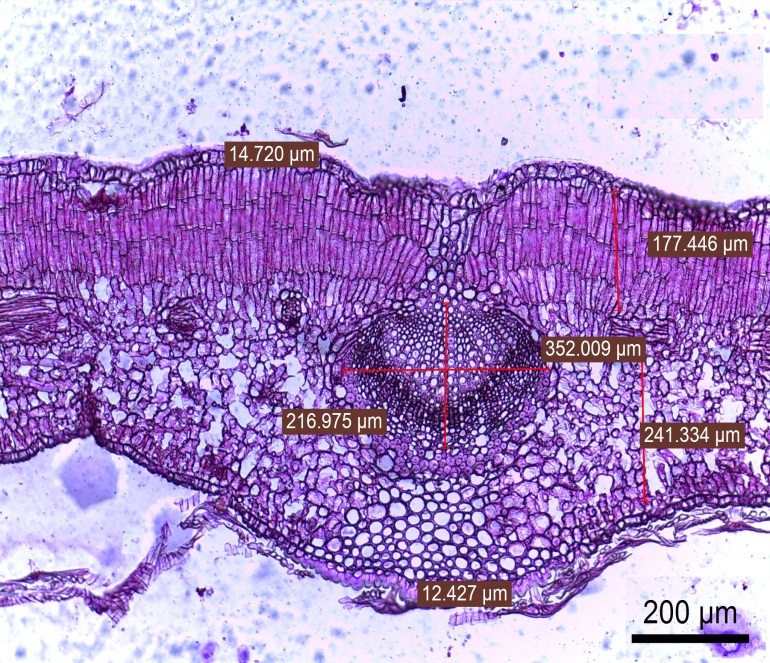 | 8 | 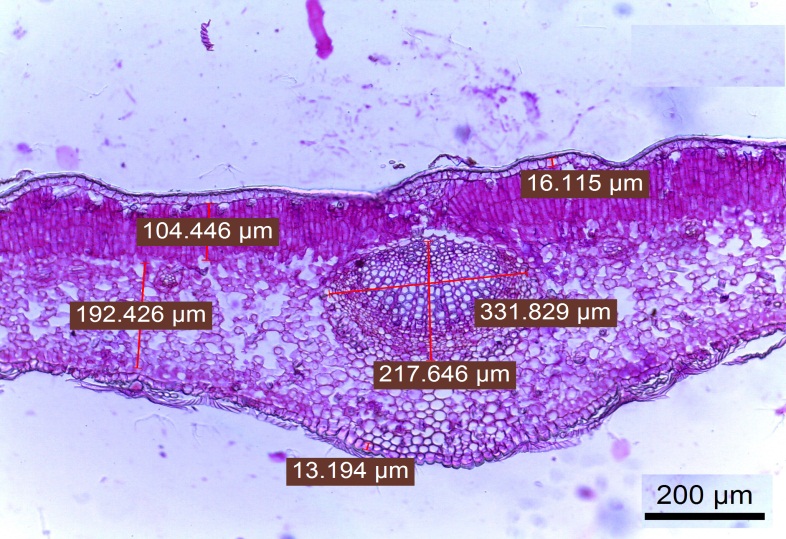 | 4 |

Figure 7: Leaf anatomical structure of different olive cultivars.


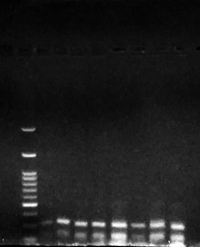


SCoT origin gel: SCoT 2


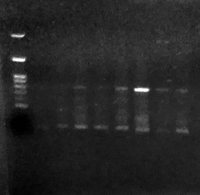


SCoT origin gel: SCoT 3


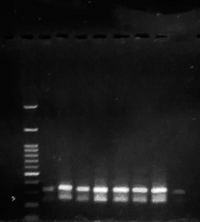

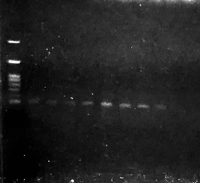
SCoT origin gel: SCoT 5

SCoT origin gel: SCoT 7


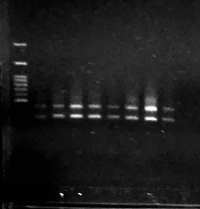


SCoT origin gel: SCoT 8


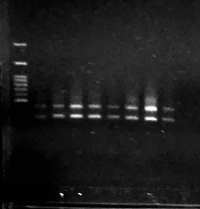


SCoT origin gel: SCoT 10


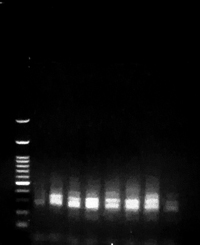


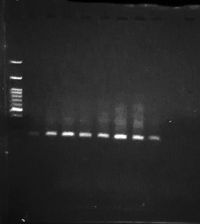
SCoT origin gel: SCoT 12

SCoT origin gel: SCoT 15

| **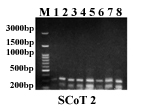** | **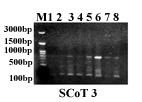** | **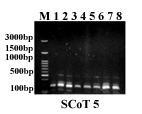** | **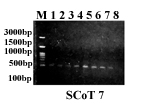** |
| --- | --- | --- | --- |
| **SCoT-2** | **SCoT-3** | **SCoT-5** | **SCoT-7** |
| **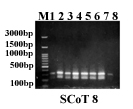** | **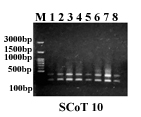** | **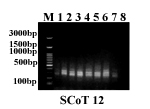** | **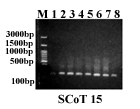** |
| **SCoT-8** | **SCoT-8** | **SCoT-12** | **SCoT-15** |

**Figure (9): Banding patterns of SCoT -PCR products for eight sOlea europea cultivars produced with eight primers.**

| **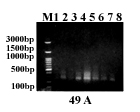** | **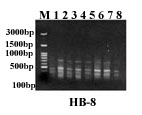** | **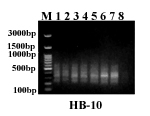** |
| --- | --- | --- |
| **49A** | **HB-8** | **HB-10** |
| **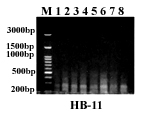** | **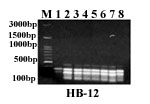** | **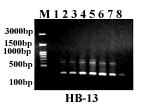** |
| **HB-11** | **HB-12** | **HB-13** |

**Figure (11): Banding patterns of ISSR -PCR products for six Olea europea cultivars produced with eight primers.**

**ISSR origin gels PIC**


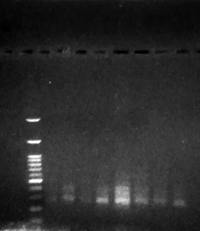


ISSR gel: 49A


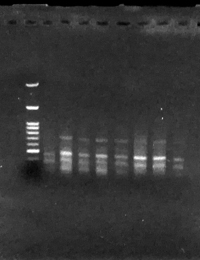


ISSR gel: HB-8


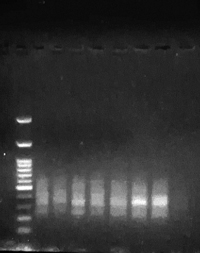


ISSR gel: HB-10


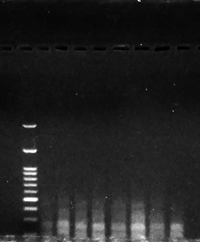


ISSR gel: HB-11


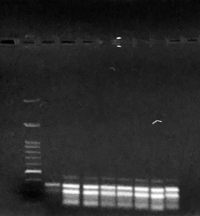


ISSR gel: HB-12


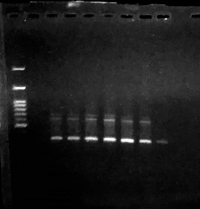


ISSR gel: HB-13
